# Relationship of the Morphology and Size of Sella Turcica with Dental Anomalies and Skeletal Malocclusions

**DOI:** 10.3390/diagnostics13193088

**Published:** 2023-09-29

**Authors:** Omid Mortezai, Haniyeh Rahimi, Maryam Tofangchiha, Sina Radfar, Mehdi Ranjbaran, Francesco Pagnoni, Rodolfo Reda, Luca Testarelli

**Affiliations:** 1 Department of Orthodontics, School of Dentistry, Qazvin University of Medical Sciences, Qazvin 34199-15315, Iran; omid_mortezai@yahoo.com; 2 Department of Orthodontics, Dental Caries Prevention Research Center, Qazvin University of Medical Sciences, Qazvin 34199-15315, Iran; 3 Department of Oral and Maxillofacial Radiology, Dental Caries Prevention Research Center, Qazvin University of Medical Sciences, Qazvin 34199-15315, Iran; 4 Department of Endodontics, Faculty of Dentistry, Tabriz University of Medical Sciences, Tabriz 51666-53431, Iran; sinarad121@gmail.com; 5 Metabolic Diseases Research Center, Research Institute for Prevention of Non-Communicable Diseases, Qazvin University of Medical Sciences, Qazvin 34199-15315, Iran; mehdiranjbaran90@yahoo.com; 6 Department of Oral and Maxillo-Facial Science, Sapienza University of Rome, Via Caserta 06, 00161 Rome, Italy; francesco.pagnoni22@gmail.com (F.P.); rodolfo.reda@uniroma1.it (R.R.); luca.testarelli@uniroma1.it (L.T.)

**Keywords:** malocclusion, sella turcica, dental anomaly

## Abstract

This study aimed to assess the relationship of the morphology and size of the sella turcica (ST) with dental anomalies and skeletal malocclusions. This cross-sectional study was conducted on records of fixed orthodontic patients treated between 2013 and 2022. Cephalometric analysis was performed to determine the anteroposterior and vertical skeletal patterns. Preoperative panoramic radiographs and lateral cephalograms, intraoral photographs, and primary dental casts of patients were used to detect dental anomalies. Gender, sagittal and vertical skeletal patterns, dental anomalies, and dimensions (length, depth, and diameter), and morphology of the ST were all recorded according to the lateral cephalograms of patients. Data were analyzed using independent t-test, one-way and two-way ANOVA, Chi-square test, and log rank test (alpha = 0.05). The depth and diameter of the ST had no significant correlation with gender (*p* > 0.05); however, the length of the ST was significantly longer in males than females (*p* < 0.05). The morphology of the ST had a significant correlation with gender (*p* < 0.05). The ST morphology had a significant correlation with the anteroposterior skeletal pattern, microdontia, and tooth impaction as well (*p* < 0.05). The present results revealed a significant correlation of the ST morphology with the anteroposterior skeletal pattern, microdontia, and tooth impaction.

## 1. Introduction

Malocclusion is a multifactorial developmental disorder, which is influenced by genetics as well as environmental and ethnic factors [1]. Any discrepancy between the skeletal position of the maxilla and mandible can lead to anteroposterior or vertical malocclusion [2]. Early detection of skeletal malocclusion is imperative for easier and more conservative treatment [3]. Many parameters have been suggested for the prediction of the growth pattern and development of malocclusion, such as the craniofacial angle, frontal sinus, and antegonial notch [4,5,6].

The sella turcica (ST) is a saddle-shaped bony structure that is anatomically located over the intracranial surface of the body of the sphenoid bone. It has two anterior clinoid processes, tuberculum sellae, hypophyseal fossa coated with diaphragm sellae, and two posterior processes. The pituitary gland disorders can change the ST morphology [7]. The ST morphology does not change significantly after 12 years of age. At 5 years of age, the anterior wall of the ST is stabilized [8]. Anomalies of the anterior wall of the ST are correlated with frontonasal anomalies, while anomalies of the posterior border may be correlated with cerebral anomalies [7].

Facial structures follow the developmental pattern of the ST [9]. Development of the midface including the ST and the teeth may change via impairment of the signaling pathways following mutations in the homeobox genes [10]. Also, endocrine disorders may affect tooth development due to functional disorders and alterations in hormonal levels.

Finding a correlation between the ST dimensions and morphology with skeletal malocclusions may help in their early detection and initiation of preventive interventions as well as decrease the future need for more complex procedures [11].

Prediction of the facial growth pattern and direction prior to the growth spurt period and puberty can be valuable [4]. Considering the cephalocaudal growth gradient, the ST dimensions may serve as a major diagnostic factor for Class II and Class III malocclusions and primary treatment planning. Since maturation of the ST occurs earlier than maturation of the mandible, the ST dimensions may help in the detection of micrognathia and macrognathia [12,13,14].

The structure of the ST can be precisely evaluated on lateral cephalograms. The ST is routinely traced in cephalometric tracing. The sella or S point at the center of the ST is a highly important reference point in cephalometric analysis [13] and evaluation of the skull morphology and intermaxillary relationship [15]. The ST can also be used for the evaluation of growth and developmental changes in orthodontic treatment.

The correlation of ST morphology and dimensions with anteroposterior [11,16,17] and vertical [18] skeletal malocclusion has been previously investigated. Also, some studies assessed the correlation of the presence of ST bridge with dental anomalies [14,19,20]. However, no comprehensive study has assessed the correlation of different types of ST morphology and dimensions with dental anomalies and skeletal malocclusions [21,22]. Thus, this study aimed to assess the relationship of the morphology and size of the ST with dental anomalies and skeletal malocclusions.

## 2. Materials and Methods

This study was conducted using records of eligible fixed orthodontic patients treated between 2013 and 2022, which were retrieved from the archives of the Orthodontics Department of School of Dentistry, Qazvin University of Medical Sciences. The study protocol was approved by the ethics committee of the university (IR.QUMS.REC.1401.092).

### 2.1. Sample Size

The sample size was calculated to be 450 for the assessment of the correlation of ST dimensions and morphology with dental anomalies according to a previous study [18], considering the general prevalence of dental anomalies to be 25% [23] and assuming alpha = 0.05 (Z = 1.96) and an accuracy of 4%. The required sample size for the assessment of the correlation of ST length and depth with the type of malocclusion was calculated to be 321 according to a previous study [24], using G*Power version 3.1.9.2 software and one-way ANOVA analysis, assuming alpha = 0.05 (Z = 1.96), a study power of 0.90, and an effect size of 0.20. To increase the power of the study, 550 records were included.

### 2.2. Eligibility Criteria

The inclusion criteria were being aged between 13 and 40 years [8], no history of previous orthodontic or orthopedic treatment, no history of trauma to the skull or face, no craniofacial disorders, and the absence of neurological disorders, systemic diseases, or malignancies requiring radiotherapy of the skull or face.

The exclusion criteria were lateral cephalograms with no diagnostic value, poor visualization of the ST, incorrect head position, and incomplete patient records.

### 2.3. Data Collection

After obtaining written informed consent from the patients, the dental records of 550 orthodontic patients were evaluated. Preoperative lateral cephalograms of patients were extracted from their records. All radiographs were taken with a Rayscan alpha scanner (Ray Co., Ltd., Hwaseong-si, Republic of Korea) with the exposure settings of 80 kVp, 11 mA, and 11 s time. All cephalograms were taken with the patients’ heads in natural head positions [25], with relaxed lips, and teeth in occlusion. The magnification of the device was 2.2 mm, which was taken into account in the measurements. All radiographs were digitized with a DF-Angell 880 X-ray film scanner, with 1 EF magnification and 875 dpi resolution, and were saved in TIF, which is a lossless save file format. The digital file of the radiographs was then transferred to the Scanora version 2.6.2.1 software, which supports cephalometric analyses. The related anatomical landmarks and ST morphology were evaluated and marked by an orthodontist and an oral radiologist with 95% inter-observer agreement. In case of a disagreement regarding an anatomical landmark location, another experienced oral radiologist was consulted to make a decision. Next, a trained post-graduate student of orthodontics measured the ST dimensions.

### 2.4. Assessment of ST Morphology

**Figure 1:**
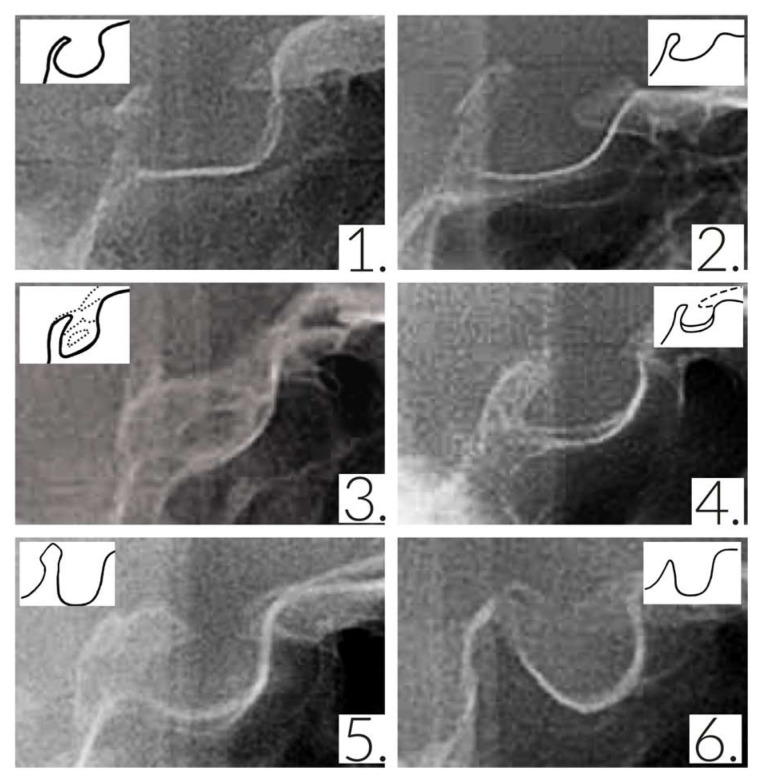
ST morphological types: (**1**) normal, (**2**) oblique anterior wall, (**3**) ST bridge, (**4**) double contour of the floor, (**5**) irregularity of the dorsum sellae, and (**6**) pyramidal-shaped dorsum sellae.

The ST morphology was categorized into six groups of normal, oblique anterior wall, ST bridge, double contour of the floor, irregularity in the dorsum sellae (notching), and pyramidal-shaped dorsum sellae (Figure 1) [26].

### 2.5. Assessment of ST Dimensions

**Figure 2:**
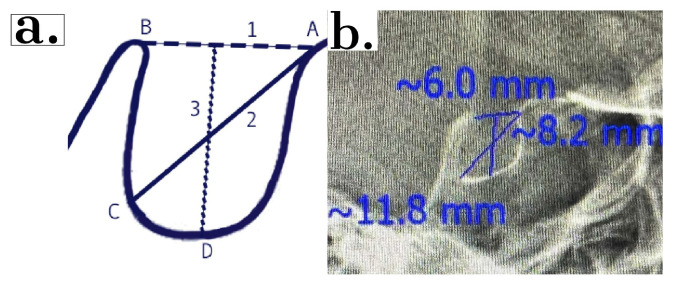
(**a**) Reference lines used for measurement of ST size: A, tuberculum sellae; B, dorsum sellae; C, the furthest point to dorsum sellae; and D, base of the pituitary fossa. 1, length of ST; 2, ST diameter; and 3, ST depth. (**b**) Scanora version 2.6.2.1 software environment.

The distance between the tuberculum sellae and dorsum sellae was recorded as the ST length. The largest anteroposterior distance of ST between the tuberculum sellae tip and the posterior wall was recorded as the ST diameter, and the depth at the deepest point of the ST floor was measured as the ST depth (Figure 2) [27,28].

### 2.6. Assessment of Anteroposterior and Vertical Skeletal Patterns

Cephalometric analysis was performed to determine the anteroposterior and vertical skeletal patterns. The anteroposterior skeletal pattern was determined according to the ANB angle. The ANB angle was drawn by identifying point A (anterior limit of the apical base of the maxilla), N (nasion), and B (anterior limit of the apical base of the mandible). The ANB values between 0 and 4 degrees indicated skeletal Class I, values > 4 degrees indicated skeletal Class II, and values < 9 indicated skeletal Class III [29].

To determine the vertical facial pattern, the patients were categorized into three groups by measuring the SN-MP angle. Those with SN-MP angle (formed between Me-Go and N-S) < 27 degrees were assigned to the short face, those with SN-MP angle between 27 and 37 degrees were assigned to the normal face, and those with SN-MP angle > 37 degrees were assigned to the long face group [30].

### 2.7. Dental Anomaly

Preoperative panoramic and cephalometric radiographs, intraoral photographs, and preoperative dental casts of patients were used for detection of dental anomalies. Other records such as cone-beam computed tomography scans of patients with palatally-impacted canine teeth were also used, if available.

Gender, anteroposterior and vertical skeletal patterns, dental anomaly, and ST dimensions and morphology were all recorded.

### 2.8. Statistical Analysis

Data were analyzed using SPSS version 25 (IBM Co., Armonk, NY, USA). The homogeneity of the variances was assessed and confirmed with the Levene’s test, and the normality of data distribution was analyzed and confirmed with a histogram and Q-Q plot. Thus, quantitative variables were compared between the two groups using independent t-test and more than two groups using one-way ANOVA and multiple ANOVA (MANOVA). Qualitative variables were compared between two or more groups with the Chi-square test and the log rank test. Pairwise comparisons were carried out using post hoc tests. The level of statistical significance was set at 0.05.

## 3. Results

Records of 550 patients including 376 females (68.4%) and 174 males (31.6%) were evaluated. The mean age of patients was 19 years (range 13 to 28 years).

### 3.1. Vertical Skeletal Pattern

Of all patients, 9.5% (n = 52) were short face, 57.5% (n = 316) were normal, and 33.1% (n = 182) were long face.

### 3.2. Anteroposterior Skeletal Pattern

Also, 42.2% (n = 232) were Class I, 43.6% (n = 240) were Class II, and 14.2% (n = 78) were Class III.

### 3.3. ST Dimensions

Table 1 presents the measures of central dispersion for the length, depth, and diameter of the ST.

### 3.4. ST Morphology

Of all patients, the ST morphology was normal in 48.4% (n = 266), ST bridge in 20.4% (n = 112), oblique anterior wall in 12% (n = 66), irregularity in the dorsum sellae in 8% (n = 44), pyramidal-shaped dorsum sellae in 6.4% (n = 35), and double contour of the floor in 4.9% (n = 27).

### 3.5. Dental Anomaly

Of all patients, 52.4% (n = 288) did not have any dental anomaly, 14.9% (n = 82) had hypodontia, 14% (n = 77) had impaction, 4.9% (n = 27) had microdontia, 3.8% (n = 21) had hyperdontia, and 3.5% (n = 19) had transposition.

### 3.6. Correlation of the ST Morphology with Anteroposterior Skeletal Pattern

Table 2 presents the correlation of the ST morphology with the anteroposterior skeletal pattern.

The Chi-square test showed a significant correlation between the ST morphology and anteroposterior skeletal pattern (*p* = 0.009), such that the normal morphology of ST had a significantly higher frequency in Class I and Class II patients; while, ST bridge, irregularity in the dorsum sellae, and pyramidal-shaped dorsum sellae had a higher frequency in Class III individuals. Double contour of the floor had a higher frequency in Class I cases.

### 3.7. Correlation of the ST Dimensions with Anteroposterior Skeletal Pattern

Table 3 presents the correlation of the ST dimensions with the anteroposterior skeletal pattern.

Although the mean dimensions of the ST in Class I patients were higher than the corresponding values in Class II and Class III cases, no significant correlation was found between the ST dimensions and anteroposterior skeletal pattern (*p* > 0.05).

### 3.8. Correlation of the ST Morphology with Vertical Skeletal Pattern

Table 4 presents the correlation of the ST morphology with the vertical skeletal pattern.

### 3.9. Correlation of the ST Dimensions with Vertical Skeletal Pattern

Table 5 presents the correlation of the ST dimensions with the vertical skeletal pattern.

### 3.10. Correlation of the ST Morphology and Dental Anomaly

Table 6 shows the correlation of the ST morphology and dental anomalies.

The Chi-square test showed significant correlations between the ST morphology and absence of dental anomaly, microdontia, and impaction (*p* < 0.05). However, the ST morphology had no significant correlation with hyperdontia, transposition, hypodontia, and third molar missing (*p* > 0.05).

### 3.11. Correlation of the ST Dimensions and Dental Anomalies

As shown in Table 7, the Chi-square test showed no significant correlation between the ST dimensions and dental anomalies.

### 3.12. Correlation of the ST Morphology and Gender

Table 8 presents the frequency of different ST morphologies based on gender.

The Chi-square test showed a significant correlation between the ST morphology and gender (*p* = 0.028), such that the prevalence of normal morphology, ST bridge, and double contour of the floor was significantly higher in females, while the prevalence of irregularity in the dorsum sellae and pyramidal-shaped dorsum sellae was higher in males.

### 3.13. Correlation of the ST Dimensions and Gender

As shown in Table 9, the independent *t*-test showed no significant correlation between the depth and diameter of the ST with gender (*p* > 0.05).

However, the ST length had a significant correlation with gender (*p* < 0.05), such that the mean ST length in males was significantly higher than that in females.

## 4. Discussion

This study assessed the relationship of the morphology and size of the ST with dental anomalies and skeletal malocclusions. The results showed a significant correlation between the ST morphology and gender, such that the prevalence of normal morphology, ST bridge, and double contour of the floor was higher in females than males, while the prevalence of irregularity in the dorsum sellae and pyramidal-shaped dorsum sellae was higher in males than females. Consistent with the present results, Motwani et al. [24] showed a significant correlation between the ST morphology and gender such that the anterior oblique wall, ST bridge, double contour of the floor, and pyramidal-shaped dorsum sellae were more commonly seen in females than males. Yan et al. [31], Bassey et al. [32], and Shrestha et al. [33] found no significant correlation between the ST morphology and gender, while Sathyanarayana et al. [9] demonstrated that anterior oblique wall, ST bridge, double contour of the floor and pyramidal-shaped dorsum sellae were more common in males than females. Variations in the results can be due to differences in the classification systems used, inclusion criteria, and methods of measurement of the dimensions and assessment of the morphology of ST.

The present results revealed a significant correlation between the ST morphology and anteroposterior skeletal pattern, such that the normal ST morphology was more commonly seen in Class I and Class II patients; however, ST bridge, irregularity in the dorsum sellae, and pyramidal-shaped dorsum sellae were more commonly detected in Class III, and double contour of the floor was more commonly seen in Class I patients. Consistent with the present results, Valizadeh et al. [34] found a significant correlation between the ST morphology and anteroposterior skeletal pattern, such that normal ST morphology was more commonly seen in Class I patients, while ST bridge, irregularity in the dorsum sellae, and pyramidal-shaped dorsum sellae were more common in Class III individuals. Sathyanarayana et al. [9] demonstrated that the ST morphology was normal in the majority (61%) of the cases, and ST bridge was more common in Class III individuals. In contrast to the present results, Al-Mohana et al. [35] found no significant correlation between the ST morphology and anteroposterior skeletal pattern.

Two reasons have been proposed for the higher incidence of ST bridge compared with other abnormal morphologies. The first reason is that the fusion between the clinoid processes may only be a radiographic finding due to superimposition of structures and not an actual bony connection. The second reason is that among different abnormal morphologies of the ST, only the ST bridge can occur during the prenatal period [12]. Also, variations in the results can be due to differences in the ST morphology and skeletal patterns in different races.

The present study found no significant correlation between the ST morphology and vertical skeletal pattern. Consistent with the present results, Afzal and Fida [11] and Ahmad et al. [18] showed no significant correlation between the ST morphology and vertical skeletal pattern. However, Yan et al. [31] and Atilla et al. [36] indicated a significant correlation between the ST morphology and vertical skeletal pattern. Variations in the reported results can be attributed to evaluation of different age groups, statistical populations, races, different methods of assessment of ST morphology, and the classification systems applied.

The current results revealed significant correlations between the ST morphology with microdontia and impaction, such that these dental anomalies were more common in individuals with ST bridge. However, no other significant correlations were found. Consistent with the present results, Jankowski et al. [21], Kaya et al. [37], Karaman et al. [38], and Jankowski et al. [39] demonstrated a significant correlation between dental anomalies and ST bridge. Alam and Alfawzan [40] indicated a higher frequency of ST bridge in patients with cleft. The majority of patients with cleft had severe skeletal Class III malocclusion associated with different dental anomalies such as impacted canine teeth, congenital missing, and presence of supernumeraries. However, Leonardi et al. [41] revealed a significantly higher frequency of ST bridging in patients with dental transposition than the control group. Since the ST mainly forms during the prenatal period (similar to most cranial base structures), different anomalies can affect the ST morphology and dimensions [12].

The present results indicated no significant correlation between the ST depth and diameter with gender; however, the ST length was significantly greater in males than females. In line with the present results, Bassey et al. [32] found a significant correlation between the length and depth of ST with gender and demonstrated that the ST length was greater in males. Motwani et al. [24] found no significant correlation between the ST depth and diameter with gender; however, the ST length was significantly greater in males than females. Sathyanarayana et al. [9] found no significant correlation between the ST depth and diameter with gender, but the ST length was significantly greater in males than females. Also, Yan et al. [31], Shrestha et al. [33], Islam et al. [42], Olubunmi et al. [43], Valizadeh et al. [34], and Nagaraj et al. [44] found no significant correlation between the ST dimensions and gender. Hasan et al. [45] indicated no significant difference between males and females in ST dimensions, except in ST depth. Variations in the reported results in the literature in this respect can be due to using different classification systems, measurement methods, and inclusion criteria.

The present results revealed no significant correlation between the ST dimensions and anteroposterior skeletal pattern. In agreement with the present results, Nadim [46] found no significant correlation between the ST dimensions and anteroposterior skeletal pattern. Also, Valizadeh et al. [34] indicated no significant correlation between the ST dimensions and anteroposterior skeletal pattern. However, Shrestha et al. [33] found a significant correlation between the ST length and diameter with anteroposterior skeletal pattern; nonetheless, the ST depth had no significant correlation with anteroposterior skeletal pattern. Moslemzadeh et al. [47] demonstrated greater ST length in Class III patients, compared with Class I and Class II cases. Sathyanarayana et al. [9] showed a significant correlation between the ST length and diameter with anteroposterior skeletal pattern, such that the ST length and diameter were greater in Class III compared with Class I and II individuals. However, the ST depth had no significant correlation with anteroposterior skeletal pattern. Variations in the results can be due to using different classification systems, radiographic techniques, and radiographic magnifications.

The present findings revealed no significant correlation between the ST dimensions and vertical skeletal pattern. The same result was reported by Taghiloo et al. [48]. Afzal and Fida [11] reported a significant difference in ST length and depth among patients with different vertical skeletal patterns; however, the ST diameter had no significant correlation with the vertical skeletal pattern. Ahmad et al. [18] found no significant correlation between the ST length and depth with vertical skeletal pattern; however, the ST diameter was significantly correlated with the vertical skeletal pattern. Variations in the results can be due to using different classification systems, radiographic techniques, and radiographic magnifications.

In the present study, no significant correlation was found between the ST dimensions and dental anomalies, which is in agreement with the results of Motwani et al. [22], and Kaya et al. [37]. Since a major part of the ST forms during the prenatal period, similar to many other cranial base structures, dental anomalies have no significant effect on its dimensions.

## 5. Conclusions

Considering the limitations of the present study, the results showed significant correlation of the ST morphology with the anteroposterior skeletal pattern, microdontia, and tooth impaction. No significant correlations were found between the ST dimensions and anteroposterior skeletal pattern, ST dimensions, and morphology with vertical skeletal pattern, or ST dimensions and dental anomalies.

## Figures and Tables

**Table 1:** Measures of central dispersion for the length, depth, and diameter of the ST in millimeters (n = 550).

Variable	Minimum	Maximum	Mean	Std. Deviation
Length	0	12	6.81	2.49
Depth	3.5	13	7.26	1.53
Diameter	6	16	10.37	1.77

**Table 2:** Correlation of the ST morphology with anteroposterior skeletal pattern.

Variable		ST Morphology						*p*-Value
		Normal	Oblique Anterior Wall	ST Bridge	Double Floor Contour	Irregular Dorsum Sellae	Pyramidal Dorsum Sellae	
**Class I**	Number	117	30	39	18	16	12	0.009
	Percentage	50.4	12.9	16.8	7.8	6.9	5.2	
**Class II**	Number	122	28	51	8	16	15	
	Percentage	50.8	11.7	21.3	3.3	6.7	6.3	
**Class III**	Number	27	8	22	1	12	8	
	Percentage	34.6	10.3	28.2	1.3	15.4	10.3	

**Table 3:** Correlation of the ST dimensions with anteroposterior skeletal pattern.

ST Dimensions	Anteroposterior Skeletal Pattern	Mean	Std. Deviation	Minimum	Maximum	*p*-Value
**Length**	Class I	7.06	2.37	0	12	0.128
	Class II	6.62	2.49	0	12	
	Class III	6.65	2.79	0	12	
**Depth**	Class I	7.32	1.50	4.5	13	0.677
	Class II	7.22	1.63	3.5	13	
	Class III	7.17	1.28	5	11	
**Diameter**	Class I	10.49	1.77	6	16	0.180
	Class II	10.36	1.73	6	15	
	Class III	10.06	1.89	6	15	

**Table 4:** Correlation of the ST morphology with vertical skeletal pattern.

Variable		ST Morphology						*p*-Value
		Normal	Oblique Anterior Wall	ST Bridge	Double Contour of Floor	Irregularity in the Dorsum Sellae	Pyramidal-Shaped Dorsum Sellae	
**Short face**	Number	22	6	15	1	5	3	0.466
	Percentage	42.3	11.5	28.8	1.9	9.6	5.8	
**Normal**	Number	163	38	56	16	20	23	
	Percentage	51.6	12	17.7	5.1	6.3	7.3	
**Long face**	Number	81	22	41	10	19	9	
	Percentage	44.5	12.1	22.5	5.5	10.4	4.9	
**Total**	Number	266	66	112	27	44	35	
	Percentage	48.4	12	20.4	4.9	8	6.4	

The Chi-square test showed no significant correlation between the ST morphology and the vertical skeletal pattern (*p* > 0.05).

**Table 5:** Correlation of the ST dimensions with vertical skeletal pattern.

ST Dimensions	Vertical Facial Form	Mean	Std. Deviation	Minimum	Maximum	*p*-Value
**Length**	Short face	7.09	2.93	1	12	0.644
	Normal face	6.82	2.48	0	12	
	Long face	6.72	2.36	0	12	
**Depth**	Short face	7.41	1.64	4.5	13	0.450
	Normal face	7.19	1.52	4	13	
	Long face	7.33	1.51	3.5	13	
**Diameter**	Short face	10.99	2.02	6	15	0.18
	Normal face	10.37	1.67	6	15	
	Long face	10.20	1.84	6	16	

One-way ANOVA showed no significant correlation in this regard (*p* > 0.05).

**Table 6:** Correlation of the ST morphology and dental anomalies.

Dental Anomaly		ST Morphology						*p*-Value
		Normal	Oblique Anterior Wall	ST Bridge	Double Contour of Floor	Irregularity in the Dorsum Sellae	Pyramidal-Shaped Dorsum Sellae	
**No anomaly**	Number	178	30	27	12	23	18	0.001
	Percentage	61.8	10.4	9.4	4.2	8	6.3	
**Hyperdontia**	Number	10	3	6	1	0	1	0.764
	Percentage	47.6	14.3	28.6	4.8	0	4.8	
**Microdontia**	Number	4	8	10	0	1	4	0.01
	Percentage	14.8	29.6	37	0	3.7	14.8	
**Impaction**	Number	20	5	30	5	12	5	0.001
	Percentage	26	6.5	39	6.5	15.6	6.5	
**Transposition**	Number	7	4	5	1	1	1	0.78
	Percentage	36.8	21.1	26.3	5.3	5.3	5.3	
**Hypodontia**	Number	13	4	12	2	2	3	0.42
	Percentage	36.1	11.1	33.3	5.6	5.6	8.3	
**Third molar missing**	Number	34	12	22	6	5	3	0.029
	Percentage	41.5	14.6	26.8	7.3	6.1	3.7	

**Table 7:** Correlation of the ST dimensions and dental anomalies.

Variable		Mean	Std. Deviation	Minimum	Maximum	*p*-Value
ST length	No anomaly	7.010	2.2708	1	12	0.474
	Hyperdontia	6.310	2.7041	0	12	
	Microdontia	6.481	3.0047	0	11	
	Impaction	6.396	2.8508	0	12	
	Transposition	6.711	2.8786	2.5	11.5	
	Missing	6.917	2.3770	1	11	
	Third molar missing	6.701	2.5782	1.5	12	
ST depth	No anomaly	7.230	1.5132	4	13	0.500
	Hyperdontia	6.881	1.7742	3.5	10	
	Microdontia	6.926	1.4787	4.5	10	
	Impaction	7.448	1.6575	4	12	
	Transposition	6.974	1.4383	5	10	
	Missing	7.431	1.4099	4	10	
	Third molar missing	7.366	1.4846	5	13	
ST diameter	No anomaly	10.411	1.7290	6	15	0.657
	Hyperdontia	10.405	2.0834	6	15	
	Microdontia	10.278	1.2810	8	12	
	Impaction	10.364	1.9912	6	15	
	Transposition	10.079	1.7342	7	13	
	Missing	10.792	1.7045	8	16	
	Third molar missing	10.140	1.8296	6	15	

**Table 8:** ST morphology based on gender.

Variable			Gender		Total	*p*-Value
			Female	Male		
ST morphology	Normal	Number	193	73	266	0.028
		Percentage	72.6	27.4	100	
	Anterior oblique wall	Number	39	27	66	
		Percentage	59.1	40.9	100	
	ST bridge	Number	79	33	112	
		Percentage	70.5	29.5	100	
	Double contour of floor	Number	21	6	27	
		Percentage	77.8	22.2	100	
	Irregularity of the dorsum sellae	Number	26	18	44	
		Percentage	59.1	40.9	100	
	Pyramidal-shaped dorsum sellae	Number	18	17	35	
		Percentage	51.4	48.6	100	
Total		Number	376	174	550	
		Percentage	68.4	31.6	100	

**Table 9:** Correlation of the ST dimensions and gender.

ST Dimensions	Gender	Mean	Std. Deviation	*p*-Value
**Length**	Female	6.662	2.4515	0.042
	Male	7.126	2.5435	
**Depth**	Female	7.306	1.5486	0.267
	Male	7.150	1.4848	
**Diameter**	Female	10.375	1.7822	0.937
	Male	10.362	1.7630	

## Data Availability

The data that support the findings of this study are available from the corresponding author.
